# Psychological drivers and value perception of medical student participation in teaching evaluation

**DOI:** 10.3389/fpsyg.2026.1928277

**Published:** 2026-08-21

**Authors:** Lilai Xu, Baixiong Chen, Surong Huang

**Affiliations:** Zhuhai Campus of Zunyi Medical University, Zhuhai, Guangdong, China

**Keywords:** behavioral intention, medical students, perceived value, student evaluation of teaching (SET), theory of planned behavior (TPB)

## Abstract

**Introduction:**

Student Evaluation of Teaching (SET) is essential for educational development; however, it increasingly suffers from survey fatigue and perfunctory responses, particularly among medical students facing high academic stress and cognitive loads. Traditional behavioral theories often overlook the psychological appraisal of “cost–benefit” in students’ decision-making processes. To address this gap, this study constructed an Extended Theory of Planned Behavior incorporating Perceived Value (Extended TPB) model, introducing “Perceived Value” as a central psychological mediator to explore the cognitive and motivational mechanisms driving students’ intentions to participate in SET.

**Methods:**

A cross-sectional survey was conducted among 3,365 undergraduate medical students at a medical university in China. Data were collected using validated scales measuring attitude, subjective norm, perceived behavioral control, perceived value, and behavioral intention. Structural Equation Modeling (SEM) was utilized to test the theoretical model, and a Bootstrapping approach with 5,000 resamples was applied to examine mediating effects.

**Results:**

The Extended TPB model demonstrated an excellent fit and high explanatory power, accounting for 95.9% of the variance in SET behavioral intention. Perceived value exerted a strong positive predictive effect on behavioral intention (*β* = 0.979, *p* < 0.001). Attitude ( β = 0.498), perceived behavioral control ( β = 0.333), and subjective norm ( β = 0.141) significantly and positively influenced perceived value. Furthermore, perceived value served as a crucial partial mediator between the three TPB antecedents and behavioral intention. Notably, senior students facing clinical rotations exhibited the lowest intention to evaluate.

**Discussion:**

Perceived value acts as a critical psychological “gatekeeper” in behavioral decision-making. Stimulating intrinsic motivation (attitude) and reducing cognitive load (perceived behavioral control) are far more effective than imposing external Controlled motivation (subjective norm). To mitigate survey fatigue, educational institutions must foster “student agency” by establishing transparent closed-loop feedback systems, thereby rebuilding the perceived psychological utility of evaluations.

## Introduction

Student Evaluation of Teaching (SET) has been adopted by almost all higher education institutions globally as a primary indicator of teaching quality (Zabaleta, 2007). At the institutional level, these evaluations serve as a quality assurance mechanism (Shah and Pabel, 2019), aimed at improving degree program content (Edgar and Gibson, 2016) and assisting faculty members in enhancing their teaching methodologies to meet student needs (Winstone and Carless, 2021). In recent years, with the comprehensive shift from traditional paper-based questionnaires to online assessments, phenomena such as declining response rates and perfunctory evaluations have become increasingly severe (Nulty, 2008). As student evaluations transition to online formats, many campuses face low response rate issues, and high-quality data necessary for decision-making processes may become unattainable (Avery et al., 2006). Recent scholarship confirms that modern high-density curricula subject professional students to severe evaluation overload, triggering cognitive saturation and inducing perfunctory rating behaviors rather than reflective qualitative feedback (Pineda and Ashour, 2022; O’Donovan, 2023). Furthermore, survey length, rigid evaluation indicators, and concerns regarding anonymity often lead students to adopt compromise behaviors, such as “random scoring” (Spooren et al., 2013). When students lack perceived value in the evaluation process or experience satisfaction fatigue, their evaluative judgments default to mental heuristics and non-differentiating response styles, yielding construct-irrelevant measurement bias (Sigurdardottir et al., 2022; Allison, 2025). When response rates decline, data quality and the reliability of outcomes are inevitably weakened (Groves, 2005). The lack of behavioral intention not only causes “small sample bias” but also undermines the diagnostic and improvement functions of SET results (Macfadyen et al., 2016). Given the current low response rates in student assessments, identifying the factors influencing student participation in SET is imperative to enhance our problem-solving capacity (Al-Maamari, 2015).

Empirical research by Adams and Umbach indicated that as the number of online questionnaires students are required to complete at the end of the term proliferates, students are highly susceptible to cognitive fatigue (Adams and Umbach, 2012). In a National Survey of Student Engagement (NSSE) targeting undergraduate populations, Porter and Umbach found that students with lower SAT scores, male students, and students of color were less likely to respond (Porter and Umbach, 2006). Chen and Hoshower noted that students’ strongest motivation to participate in SET lies in their expectation to “improve future teaching quality” through their feedback; when students perceive a lack of substantive response from school administration or faculty regarding SET results (i.e., the perception of “useless feedback”), their intrinsic behavioral intention drops significantly (Chen and Hoshower, 2003). Research by Marlin (1987) showed that 32% of written comments indicated students believed teachers would not make any adjustments based on their feedback, thus viewing SET as a waste of time. El Hassan (2009) found that only half of the students believed teachers valued and adopted their feedback, while nearly 30% completely dismissed the value of their opinions. Furthermore, Kite et al. (2015) revealed that “only 43.3% of respondents believed professors would frequently or very frequently read their comments.” This widespread perception that feedback goes unvalued or unadopted is likely a major catalyst for the pervasive issue of low response rates.

In the specific context of Chinese higher medical education, this issue is further compounded. Medical students in China undergo highly condensed curricula and rigorous clinical rotations (e.g., the ‘5 + 3’ integrated medical program). Furthermore, traditional educational cultures characterized by high power distance between faculty and students often lead students to view SET as an administrative mandate rather than an opportunity for genuine dialogue. Consequently, understanding institutional policies and cultural nuances is crucial.

From a psychological perspective, the pervasive issue of “survey fatigue” and “random scoring” (Spooren et al., 2013) is not merely an administrative failure, but a manifestation of cognitive depletion. Medical students, burdened with intense academic coursework and clinical rotations, experience high cognitive load. When forced to participate in mandatory, lengthy evaluations, their behavior shifts from intrinsic participation to mere compliance or passive resistance. In health professions education across Asian contexts, clinical service pressures combined with cultural factors (such as high power distance and hierarchical supervision) heavily constrain trainees’ engagement with feedback, converting evaluation into a low-utility administrative burden unless high developmental value and anonymity are guaranteed (Fullerton et al., 2025). Therefore, understanding SET participation requires shifting the focus from administrative reinforcement to students’ internal psychological appraisals.

Spoorena and Van Loon (2012) found that students generally believe completing SETs does not enhance their academic performance; this, compounded with the high-stakes learning environments of medical students, likely contributes to low response rates. Min and Baozhi (1998) discovered significant differences in SET scores across different disciplines. For instance, SET scores in clinical science majors are generally higher than those in basic science majors. The authors attribute this to medical students’ strong desire to become physicians; thus, they possess higher personal interest and engagement motivation in clinical courses compared to basic science courses (Royal et al., 2018). Although differences exist between general higher education and medical education, the SET methods used in general higher education are often directly applied to medical and healthcare professional courses. However, implementing these general SET methods in medical education faces numerous challenges. Specifically, due to variations in teaching methods and professional expertise among different instructors, a single course evaluation may fail to capture a student’s holistic assessment of all teachers or the course itself. Research by Uijtdehaage and O’Neal (2015) suggests that requiring students to evaluate multiple instructors individually at the end of a course may be inappropriate, as students practically struggle to recall and evaluate the distinct content delivered by numerous teachers. Moreover, many faculty members in medical education are primarily clinicians, with teaching being a secondary responsibility (van Bruggen et al., 2020). Because their primary focus is patient care, and they are often required to participate in research activities, they frequently lack the time to refine their teaching skills.

The classic Theory of Planned Behavior (TPB) posits that an individual’s behavioral intention is jointly determined by attitude toward the behavior, subjective norm, and perceived behavioral control (Ajzen, 1991). However, in the specific context of higher medical education, medical students universally face extreme academic burdens and clinical practicum pressures. When attempting to explain SET participation—a behavior that often exhibits characteristics of “compromise” or “utilitarianism”—the traditional TPB model falls short by ignoring the rational trade-off individuals make between the time/energy expended and the anticipated benefits (Jia et al., 2025).

**Figure 1:**
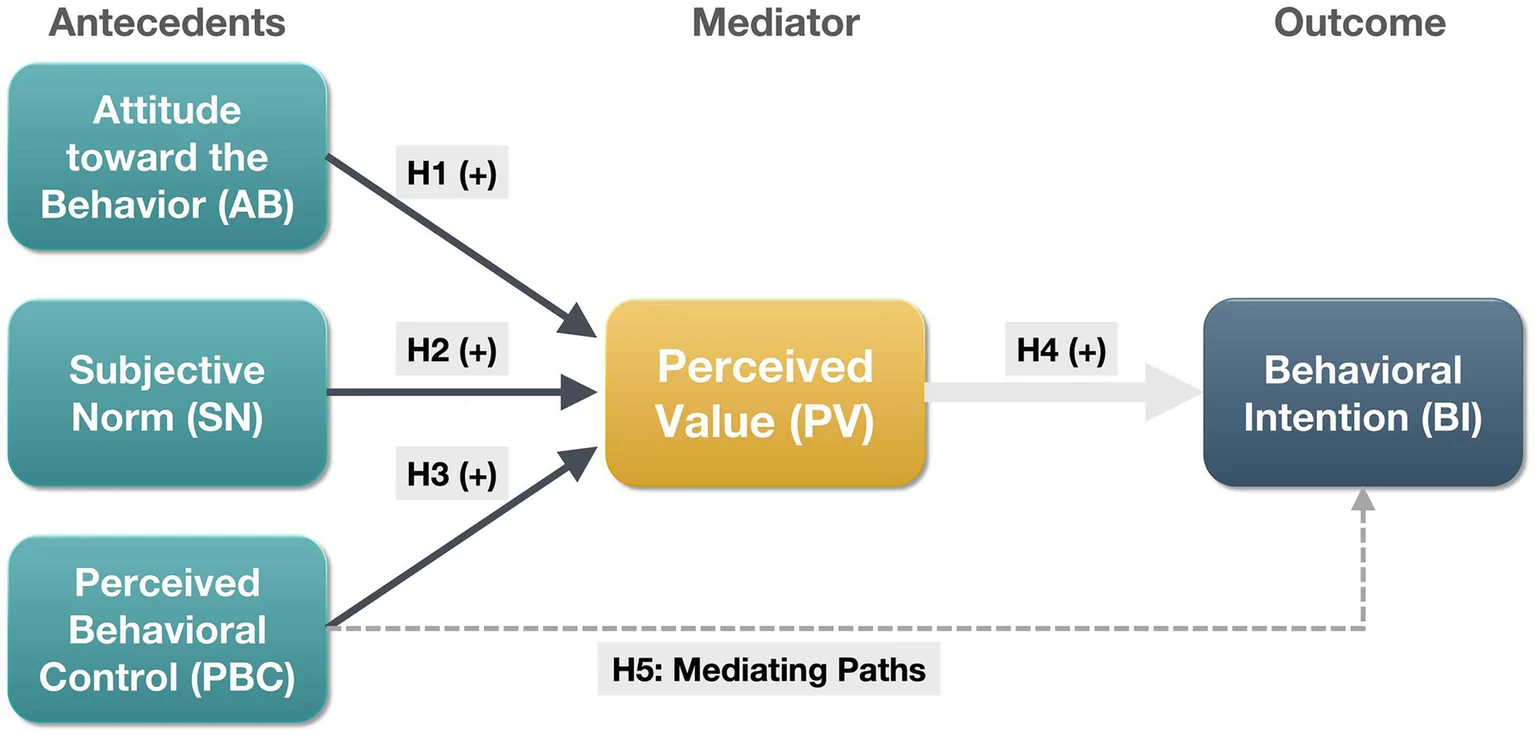
The conceptual framework of the Extended Theory of Planned Behavior incorporating Perceived Value (Extended TPB) model.

To address this gap, this study introduces the concept of “Perceived Value” to extend the traditional TPB model (Iskandar et al., 2024). While rooted in consumer behavior, integrating perceived value aligns with Expectancy-Value Theory and recent integrative TPB frameworks in educational research, which emphasize combining social-cognitive decision-making with self-determined motivational mechanisms (Fullerton et al., 2025; Jia et al., 2025). This extended model posits that in the context of medical students’ SET, attitude, subjective norm, and perceived behavioral control do not directly translate into behavioral intention; rather, perceived task value governs whether cognitive dispositions transform into authentic evaluative engagement. Specifically, these traditional TPB antecedents act as antecedent triggers that influence students’ holistic cognitive appraisal of “SET utility.” Based on cognitive appraisal theory, individuals constantly evaluate whether the environmental demands (time and effort, i.e., cognitive costs) outweigh the personal significance of the outcome (teaching improvement). This psychological “cost–benefit” trade-off, captured by “Perceived Value,” subsequently drives their ultimate behavioral intention to participate in SET (as depicted in Figure 1). Figure 1 visually illustrates the hypothesized directional paths, demonstrating how Perceived Value operates as a central psychological bridge linking the three core TPB antecedents with students’ final behavioral intentions.

## Research hypotheses

### Attitude and perceived value

Attitude refers to an individual’s positive or negative evaluation of performing a specific behavior. In the context of teaching evaluations, when students hold a positive attitude toward SET—viewing it as “meaningful” or “wise”—they are more inclined to focus on the long-term positive benefits it brings (e.g., facilitating improvements in teaching quality). Given the high-stakes nature of medical training, where clinical competence directly impacts patient safety, students who believe SET will genuinely enhance their pedagogical training are far more likely to assign high intrinsic utility to the evaluation process. This positive internal psychological predisposition can effectively enhance the individual’s value perception of evaluation activities. Thus, we hypothesize:

*H1*: Medical students’ attitude toward SET has a significant positive effect on their perceived value.

### Subjective norm and perceived value

Subjective norm reflects the perceived external social pressure an individual feels when deciding whether to perform a behavior. Within the rigorous organizational culture of medical universities, encouragement from academic advisors, widespread participation among peer groups, and institutional constraints form a powerful external push. In Asian medical education settings characterized by high power distance and Confucian work dynamism, teacher-student feedback exchanges are strongly dictated by institutional hierarchy and relational harmony, leading students to weigh normative pressure heavily when evaluating utility (Gao et al., 2025). According to Social Influence Theory, external norms not only induce compliance but can also prompt individuals to internalize the core meaning of those norms. In the highly hierarchical culture of Chinese medical education (e.g., the ‘5 + 3’ clinical residency system), trainees are deeply embedded in strict mentor-apprentice relationships. Consequently, strong institutional and supervisory expectations can heavily shape their cognitive baseline for what is deemed “valuable” compliance. When medical students perceive strong expectations to evaluate, they tend to re-examine and acknowledge the necessity of SET, thereby elevating their perception of its intrinsic value. Thus, we hypothesize:

*H2*: Medical students’ perceived subjective norm has a significant positive effect on their perceived value.

### Perceived behavioral control and perceived value

Perceived behavioral control refers to an individual’s perception of the ease or difficulty of performing a particular behavior. The core of perceived value lies in the trade-off between “Perceived Benefits (Get)” and “Perceived Costs (Give).” For medical students burdened with heavy coursework, if the SET platform is user-friendly and evaluation criteria are clear (i.e., high perceived behavioral control), the time and cognitive costs required to complete the evaluation (perceived give) are drastically reduced. Assuming anticipated benefits remain constant, a reduction in execution costs directly leads to a significant surge in net perceived value. Medical students frequently suffer from ‘time poverty’ due to grueling clinical shift rotations and intense academic curricula. Under such cognitive depletion, an intuitive, low-effort evaluation platform prevents cognitive overload, directly preserving the perceived net value of the evaluation task. Thus, we hypothesize:

*H3*: Medical students’ perceived behavioral control has a significant positive effect on their perceived value.

### Perceived value and behavioral intention

Perceived value serves as the ultimate “gatekeeper” in individual behavioral decision-making. Existing empirical research indicates that students’ strongest motivation to participate in SET is the expectation of receiving substantive feedback for teaching improvement (Chen and Hoshower, 2003). When medical students deeply perceive that SET not only benefits their own learning but is also an effective tool for driving curriculum reform, this high perceived value directly stimulates a strong, non-perfunctory, and authentic intention to evaluate. Conversely, if they fall into the “useless feedback” mindset, their intention to evaluate will severely dissipate. Thus, we hypothesize:

*H4*: Perceived value has a significant positive effect on medical students’ intention to participate in SET.

### The mediating role of perceived value

Synthesizing the above logic, this study argues that while the three core elements of traditional TPB constitute the initial driving forces and boundary conditions for medical students’ SET, these antecedent variables must pass through the psychological bridge of “perceived value” to effectively awaken genuine student agency in feedback. Perceived value plays a central transformative role between external constraints/initial attitudes and the final behavioral decision. Thus, we hypothesize:

*H5*: Perceived value mediates the relationships between attitude (H5a), subjective norm (H5b), perceived behavioral control (H5c), and intention to participate in SET.

## Methods

### Data source and sample characteristics

To capture the complex psychological mechanisms underlying SET participation, this study adopted a mixed-methods exploratory sequential design, combining qualitative case interviews with a large-scale quantitative survey. In the first phase (conducted between September and October 2025), semi-structured in-person interviews were carried out with 12 key informants at Zunyi Medical University, comprising 3 SET administrators, 3 faculty members, and 6 undergraduate medical student representatives across different academic years. The interview protocol focused on evaluating willingness to participate, perceived structural barriers, cognitive burdens during clinical coursework, and expectations regarding feedback loops. The qualitative data underwent thematic analysis, revealing that clinical rotation fatigue, anonymity concerns, and perceived feedback utility were critical determinants. These qualitative insights directly guided the conceptualization of domains and the tailored item design for the Perceived Value and Perceived Behavioral Control constructs.

In the second phase, a pilot study involving 200 medical students was conducted to evaluate item readability, factor structure, and initial reliability, following which minor phrasing adjustments were made. Subsequently, the formal quantitative survey was administered online via the university’s official academic affairs portal and mobile application during the end-of-term evaluation window (over a three-week period from November to December 2025). Participation was strictly voluntary and anonymous, with electronic informed consent obtained prior to questionnaire access. A total of 4,539 formal questionnaires were distributed. Strict data-screening protocols were applied: questionnaires exhibiting straight-lining (i.e., selecting the same option for more than 70% of the items) or missing data exceeding 70% were flagged as invalid. After excluding invalid responses, 3,365 valid questionnaires were retained for statistical analysis, yielding an effective response rate of 74.1%.

### Variables and measurement

By reviewing literature on student SET motivations and incorporating the characteristics of SET, the influencing factors of behavioral intention were categorized into three dimensions: attitude, subjective norm, and perceived behavioral control. Perceived value was introduced as a mediator, and behavioral intention served as the dependent variable. Regarding measurement, the TPB dimension scales primarily referenced mature scales from Hoel and Dahl (2019), and the perceived value scale referenced the original scale by Davis (1989), consisting of 5 items to capture anonymity, learning benefit, genuine expression, course improvement, and truthful response. Based on recommendations from SET administrators and experts, the *Medical Student Intention for SET Scale* was developed. All items were measured using a 5-point Likert scale, ranging from 1 (“Strongly Disagree”) to 5 (“Strongly Agree”). The specific measurement items for each construct, along with their corresponding descriptive statistics (means and standard deviations), are comprehensively detailed in Table 1.

**Table 1:** Construct items, descriptive statistics, and measurement items (*N* = 3,365).

Dimension	Code	Item	Mean	S.D.
Attitude toward the behavior (AB)	AB1	I think participating in SET is meaningful.	3.904	1.003
	AB2	I think SET can effectively promote teaching improvement.	3.936	0.980
	AB3	I think participating in SET is a wise behavior.	3.927	0.969
Subjective norm (SN)	SN1	I will participate in SET because of encouragement from my counselor/head teacher.	3.916	0.985
	SN2	I will participate in SET because my classmates generally do.	3.885	0.987
	SN3	The university’s SET systems (notices, requirements) prompt me to participate.	3.935	0.965
Perceived behavioral control (PBC)	PBC1	I will actively participate in SET if I have strong opinions about the course.	3.866	0.984
	PBC2	I will actively participate in SET if I have strong opinions about the instructor.	3.845	0.986
	PBC3	I understand the relevant content and specific requirements of SET.	3.824	0.971
Perceived value (PV)	PV1	I answer SET questions accurately and truthfully.	3.891	0.975
	PV2	I think SET is anonymous and will not affect my course grades.	3.867	1.033
	PV3	I think SET can benefit my learning.	3.830	0.996
	PV4	SET allows me to truly express my feelings about the course.	3.922	0.960
	PV5	SET is an effective tool for driving course improvement.	3.932	0.945
Behavior of intention (BI)	BI1	I am willing to express my satisfaction with the course through SET.	3.967	0.930
	BI2	I am willing to provide feedback for course improvement through SET.	3.968	0.928
	BI3	I usually complete SET immediately after receiving the notification.	3.906	0.987

### Reliability and validity testing

The reliability of the constructs was evaluated using Cronbach’s α and Composite Reliability (CR). Results showed that the Cronbach’s α and CR values for all five latent variables were above 0.90 (well exceeding the threshold of 0.70), indicating excellent internal consistency of the measurement model. Regarding convergent validity, Confirmatory Factor Analysis (CFA) results indicated that the Standardized Factor Loadings of all observed variables on their corresponding latent variables ranged from 0.776 to 0.966, all reaching high significance levels (*p* < 0.001). Simultaneously, the Average Variance Extracted (AVE) for all latent variables exceeded the 0.5 threshold. These metrics verify good convergent validity (Bagozzi and Yi, 1988). Discriminant validity was assessed using the Fornell and Larcker (1981) criterion. Results showed that the square root of the AVE for each latent variable (ranging from 0.892 to 0.955) was greater than the absolute correlation coefficients between that latent variable and all other latent variables. This demonstrates that the five latent variables have both theoretical correlation and good empirical distinctiveness, supporting discriminant validity. Furthermore, to rigorously rule out conceptual redundancy between Perceived Value and Behavioral Intention, Heterotrait-Monotrait (HTMT) ratios of correlations were calculated. All HTMT values between constructs ranged from 0.621 to 0.798, well below the conservative standard threshold of 0.85, confirming robust discriminant validity despite their high statistical correlation.

### Statistical analysis

All statistical analyses were conducted using IBM SPSS Statistics (Version 26.0) and IBM SPSS AMOS (Version 24.0). The analytic steps were prespecified as follows:

First, descriptive statistics were computed to summarize the demographic characteristics and the mean scores (with standard deviations, SD) of the questionnaire items. To assess demographic differences in SET intentions, independent sample t-tests and one-way Analysis of Variance (ANOVA) were performed.

Second, the measurement model was evaluated for reliability and validity. Internal consistency reliability was determined using Cronbach’s alpha (*α*) and Corrected Item-Total Correlation (CITC) values. Construct validity was assessed through Exploratory Factor Analysis (EFA), confirmed by the Kaiser-Meyer-Olkin (KMO) measure and Bartlett’s Test of Sphericity. Confirmatory Factor Analysis (CFA) was subsequently executed to evaluate convergent validity, indicated by standardized factor loadings, Average Variance Extracted (AVE), and Composite Reliability (CR). Finally, Structural Equation Modeling (SEM) was employed to test the hypothesized driving paths of the Extended TPB model. Model fit was evaluated using multiple indices, including Comparative Fit Index (CFI > 0.9), Normed Fit Index (NFI > 0.9), and Standardized Root Mean Square Residual (SRMR < 0.05). To rigorously test the mediating role of Perceived Value (PV) between the three TPB antecedents and Behavior Intention, a percentile Bootstrap estimation method with 5,000 resamples was utilized. A 95% Confidence Interval (CI) not containing zero indicated a statistically significant mediating effect. A two-sided *p*-value of <0.05 was considered statistically significant for all tests.

Prior to structural modeling, preliminary diagnostic analyses were performed to verify statistical assumptions and data quality. Skewness and kurtosis were assessed to evaluate data normality, with all values falling within the acceptable ranges (skewness < 2.0, kurtosis < 7.0). To check for potential multicollinearity among the predictor variables, Variance Inflation Factor (VIF) values were calculated; all VIFs ranged from 1.45 to 2.82, well below the conservative threshold of 5.0, indicating no severe multicollinearity. Furthermore, given the single-source and self-reported nature of the survey, Harman’s single-factor test was executed to assess Common Method Bias (CMB). The first unrotated factor accounted for 38.6% of the total variance, which is strictly below the critical 50% cutoff, confirming that CMB was not a significant threat to the validity of the results. Additionally, strict participant anonymity and neutral item phrasing were guaranteed during administration to minimize social desirability bias.

## Results

### Demographic differences analysis

Independent samples t-tests and one-way ANOVA revealed significant differences in students’ SET intention levels across different demographic characteristics. The detailed statistical comparisons, including t-values and *F*-values across categories such as gender, grade, major, and GPA, are summarized in Table 2. Specifically, significant differences were found in terms of grade, major, GPA, and student leadership experience, while no significant difference was observed for gender. Regarding grade level, juniors showed the highest SET intention (4.06), while seniors exhibited the lowest (3.82). Among majors, clinical medicine students had a significantly higher intention (3.98) than other majors, with stomatology students being the lowest (3.81). Students with an excellent GPA ( ≥ 3.5) demonstrated the highest intention (4.06). Finally, students who had served as student leaders exhibited significantly higher SET intention (4.01) compared to those who had not (3.87).

**Table 2:** Differences in behavioral intention items across demographic variables.

Variables	Categories	BI1 (Mean ± SD)	BI2 (Mean ± SD)	BI3 (Mean ± SD)
Gender	Female	3.97 ± 0.88	3.97 ± 0.88	3.91 ± 0.95
	Male	3.96 ± 1.02	3.96 ± 1.01	3.90 ± 1.05
	Test statistic (t)	0.182	0.261	0.398
Grade	Freshman	3.93 ± 0.91	3.94 ± 0.89	3.88 ± 0.94
	Sophomore	3.95 ± 0.94	3.95 ± 0.94	3.92 ± 0.99
	Junior	4.08 ± 0.91	4.09 ± 0.91	4.01 ± 0.99
	Senior	3.87 ± 0.98	3.85 ± 0.98	3.73 ± 1.05
	Test statistic (F)	6.066***	7.496***	8.173***
Major	Clinical medicine	4.01 ± 0.93	4.01 ± 0.93	3.93 ± 1.00
	Medical imaging	3.97 ± 0.88	3.97 ± 0.86	3.82 ± 1.01
	Stomatology	3.82 ± 0.95	3.82 ± 0.95	3.78 ± 0.99
	Nursing	3.93 ± 0.95	3.94 ± 0.95	3.91 ± 0.97
	Pharmacy	3.95 ± 0.87	3.97 ± 0.87	3.99 ± 0.90
	Test statistic (F)	3.197*	3.104*	2.686*
GPA	2.0–2.4	3.83 ± 1.03	3.86 ± 1.01	3.81 ± 1.05
	2.5–2.9	3.92 ± 0.92	3.90 ± 0.92	3.85 ± 0.98
	3.0–3.4	3.97 ± 0.92	3.97 ± 0.93	3.93 ± 0.95
	≥ 3.5	4.05 ± 0.89	4.06 ± 0.88	3.97 ± 0.98
	Unclear/no record	3.80 ± 1.12	3.78 ± 1.14	3.67 ± 1.18
	Test statistic (F)	4.748**	5.617***	4.052**
Student leader	Yes	4.03 ± 0.91	4.03 ± 0.90	3.96 ± 0.98
	No	3.90 ± 0.95	3.89 ± 0.95	3.84 ± 0.99
	Test statistic (t)	16.177***	17.932***	12.065***

BI1, Willingness to express satisfaction; BI2, Willingness to provide feedback for improvement; BI3, Immediate completion of evaluation. *p < 0.05, **p < 0.01, ***p < 0.001.

### Subgroup profile analysis and importance-performance prioritization

Beyond aggregate comparisons, synthesizing the empirical findings across construct performance (Table 1 means), demographic profiles (Table 2), and path impacts (Table 3) reveals vital structural heterogeneities and administrative priorities:

**Table 3:** Summary of model regression coefficients.

Structural path/measurement item	Unstandardized estimate (B)	SE	CR(z-value)	*p*-value	Standardized estimate (β)
Structural model (hypothesis paths)					
Attitude ➔ perceived value	0.417	0.018	23.026	< 0.001	0.498
Subjective norm ➔ perceived value	0.120	0.021	5.816	< 0.001	0.141
Perceived behavioral control ➔ perceived value	0.285	0.016	18.362	< 0.001	0.333
Perceived value ➔ behavioral intention	1.107	0.015	73.075	< 0.001	0.979
Measurement Model (factor loadings)					
*Attitude*					
➔ AB3 (Wise behavior)	0.996	0.008	123.222	< 0.001	0.966
➔ AB2 (Promote improvement)	1.004	0.008	121.127	< 0.001	0.962
➔ AB1 (Meaningful)	1.000	-	-	-	0.937
*Subjective Norm*					
➔ SN3 (Institutional regulations)	0.899	0.011	78.905	< 0.001	0.858
➔ SN2 (Peers’ participation)	0.998	0.010	100.462	< 0.001	0.931
➔ SN1 (Advisor’s encouragement)	1.000	-	-	-	0.935
*Perceived behavioral control (PBC)*					
➔ PBC2 (Strong opinions on instructor)	0.971	0.011	88.613	< 0.001	0.906
➔ PBC1 (Strong opinions on course)	1.000	-	-	-	0.934
➔ PBC3 (Understand requirements)	0.881	0.012	71.880	< 0.001	0.834
*Perceived value*					
➔ PV5 (Tool for curriculum improvement)	1.143	0.015	75.280	< 0.001	0.952
➔ PV4 (Express true feelings)	1.140	0.016	72.802	< 0.001	0.935
➔ PV3 (Benefit from SET)	1.165	0.016	70.835	< 0.001	0.921
➔ PV2 (Anonymity / No impact on grades)	1.019	0.019	53.842	< 0.001	0.776
➔ PV1 (Answer accurately/truthfully)	1.000	-	-	-	0.824
*Behavioral intention*					
➔ BI3 (Complete immediately)	0.948	0.011	83.695	< 0.001	0.854
➔ BI2 (Provide feedback for improvement)	1.001	0.008	133.187	< 0.001	0.960
➔ BI1 (Express satisfaction)	1.000	-	-	-	0.957

SE , Standard Error; CR , Critical Ratio (z-value). The dashes (“-”) denote parameters fixed to 1.000 to set the metric for the latent constructs, hence no standard errors or p -values are computed for these reference items.

First, Subgroup Profile Heterogeneity: A clear gradient emerges across student profiles. Senior medical trainees undergoing clinical rotations exhibited the lowest evaluation intention (mean = 3.82, *p* < 0.001), contrasting sharply with juniors (4.06). Similarly, students with high academic performance (GPA ≥ 3.5, mean = 4.06) and student leadership experience (mean = 4.01) demonstrated significantly higher engagement than their peers (*p* < 0.001).

Second, Importance-Performance Alignment: Comparing the structural impacts (*β*) with baseline construct performance reveals that Attitude holds the highest importance (*β* = 0.498) and performance (mean = 3.923). Crucially, Perceived Behavioral Control (PBC) demonstrates strong structural importance (*β* = 0.333) but yields the lowest baseline performance score (mean = 3.845) among all constructs. Conversely, Subjective Norm shows the weakest impact (*β* = 0.141) despite relatively high performance (mean = 3.912). This identifies PBC (execution ease/cost reduction) as the primary operational bottleneck for institutional intervention.

### Path analysis of the extended TPB model

**Figure 2:**
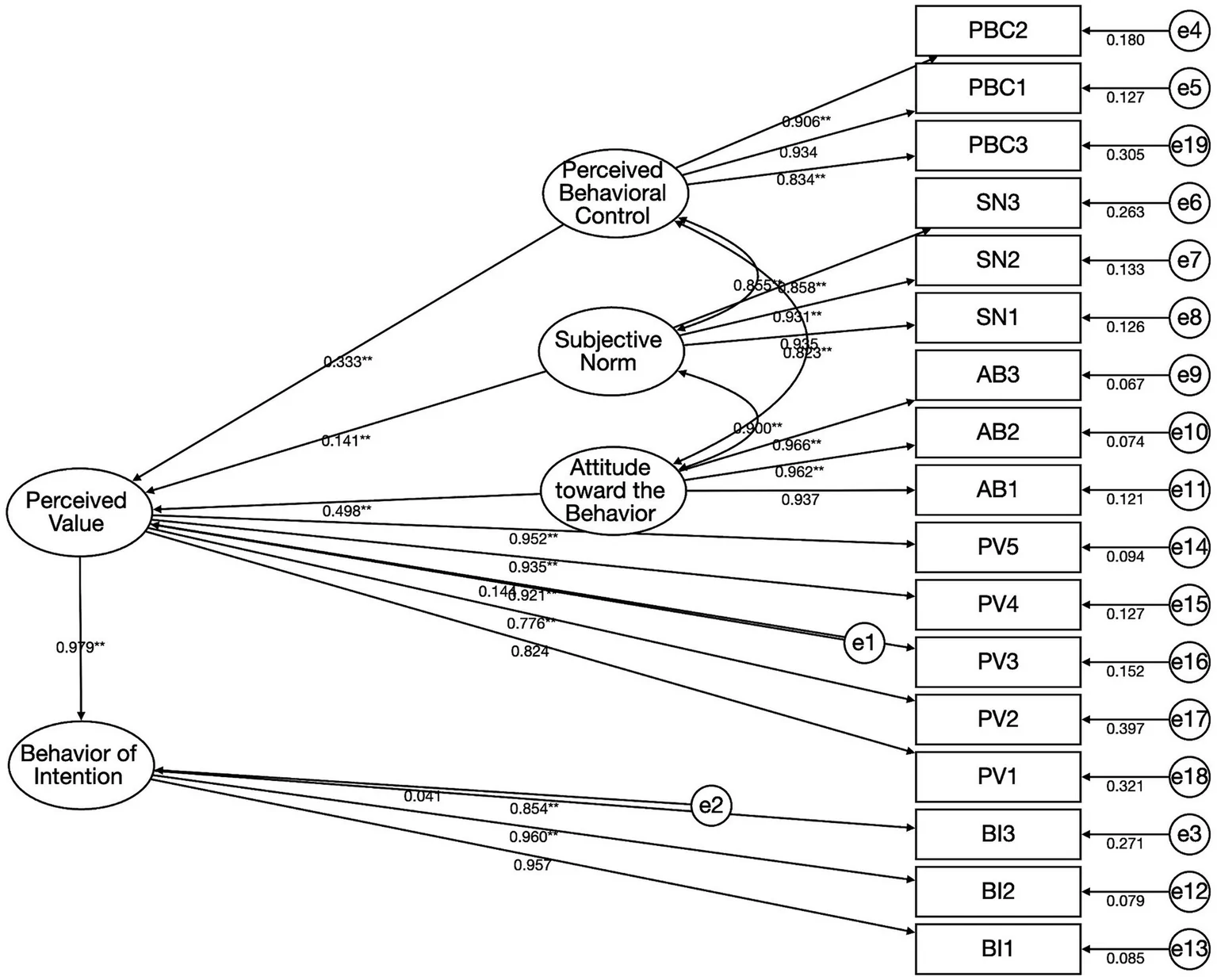
Path analysis results of the structural equation model for the Extended TPB framework, showing standardized path coefficients and factor loadings.

First, the overall validity of the structural model was evaluated. Fit indices demonstrated good model fit: the Comparative Fit Index (CFI) was 0.967 and the Tucker-Lewis Index (TLI/NNFI) was 0.960, both exceeding the 0.90 standard. The Standardized Root Mean Square Residual (SRMR) was 0.036 (well below the 0.08 threshold), and the Root Mean Square Error of Approximation (RMSEA) was 0.084, falling within an acceptable range. Although the ratio of chi-square to degrees of freedom (*χ^2^ / df* = 24.516) exceeded conventional thresholds due to the statistical sensitivity of the exceptionally large sample size (*N* = 3,365), relying on relative fit indices (CFI, TLI, SRMR) that are unaffected by absolute sample size is an established practice in large-sample research (Hair et al., 2010). Overall, the structural model demonstrated good fit and high validity, making it suitable for subsequent hypothesis testing. The complete empirical path diagram, including standardized path coefficients and measurement item loadings, is presented in Figure 2.

Under the premise of model validity, causal paths within the model were tested. Parameter estimation results revealed that the four main effect hypotheses proposed in this study were highly supported by empirical data (*p* < 0.001), as visually summarized in Figure 2. The complete summary of model regression coefficients, including unstandardized estimates, standard errors (SE), critical ratios (CR), and standardized path coefficients (β), is reported in Table 3.

The analytical results show that all three core variables of the classic TPB can effectively transform into medical students’ value perception of SET utility. Among them, attitude exhibited the strongest positive impact on perceived value (Standardized path coefficient *β* = 0.498, *p* < 0.001), supporting H1. This was followed by perceived behavioral control, which also showed a significant positive predictive effect on perceived value (*β* = 0.333, *p* < 0.001), supporting H3. Although the impact of subjective norm on perceived value was relatively smaller, it still reached a highly significant positive level (*β* = 0.141, *p* < 0.001), supporting H2. This indicates that a positive attitude toward evaluation, convenient evaluation procedures, and external administrative expectations collectively construct the psychological perception that “SET is valuable” among medical students.

Regarding the value transformation path, data revealed an extremely strong predictive association. Perceived value exerted a strong positive predictive effect on ultimate behavioral intention (*β* = 0.979, *p* < 0.001), robustly supporting H4. This finding confirms the “gatekeeper” effect of perceived value in medical students’ behavioral decision-making process: as long as students acknowledge the perceived psychological utility of SET, their genuine intention to participate will surge exponentially.

To further validate the explanatory efficacy of the extended Extended TPB model, squared multiple correlations (*R^2^*) of the endogenous latent variables were examined. Results showed that the three independent variables (attitude, subjective norm, and PBC) jointly explained 85.6% of the variance in “perceived value” (*R^2^* = 0.856); remarkably, the model’s ultimate explanatory power for the target dependent variable, “behavioral intention,” reached an impressive 95.9% (*R^2^* = 0.959). In behavioral science research, an *R^2^* greater than 0.50 is generally considered to possess strong explanatory power (Cohen, 1988). The extremely high explained variance in this model fully demonstrates that introducing “perceived value” substantially elevates the predictive precision of the traditional TPB within the medical student SET context, verifying the scientific validity and enhanced explanatory power of the extended model theoretical framework.

### The mediating effect of perceived value

A Bootstrapping resampling method (set to 5,000 iterations) was employed to rigorously test the mediating effect of perceived value (PV). As presented in Table 4, the total, direct, and indirect effects were systematically calculated, along with their 95% confidence intervals (CI), to determine the specific mediation types. The indirect effects of attitude (AB), subjective norm (SN), and perceived behavioral control (PBC) on behavioral intention (BI) via perceived value (PV) were all significantly positive, with their 95% Confidence Intervals (CI) excluding zero.

**Table 4:** Bootstrapping results for the mediating effect of perceived value.

Mediation paths	Total effect (c)	Direct effect (c’)	Indirect effect (a × b)	Boot SE	Boot 95% CI (LL, UL)	Mediation type
AB → PV → BI	0.412***	0.129**	0.284***	0.022	[0.237, 0.337]	Partial
SN → PV → BI	0.172***	0.070**	0.102***	0.016	[0.069, 0.125]	Partial
PBC → PV → BI	0.321***	0.111**	0.209***	0.021	[0.177, 0.256]	Partial

*N* = 3,365. Bootstrap sample size = 5,000. AB, Attitude toward Behavior; SN, Subjective Norm; PBC, Perceived Behavioral Control; PV, Perceived Value; BI, Behavioral Intention. Boot SE, Bootstrapping standard error. Boot 95% CI, Bootstrap 95% confidence interval (Lower Limit, Upper Limit). If the 95% CI does not include zero, the indirect effect is significant. **p < 0.01, ***p < 0.001.

Specifically, the indirect effect value for the path “Attitude ➔ PV ➔ BI” was 0.284 (95% CI [0.237, 0.337]); for “Subjective Norm ➔ PV ➔ BI,” it was 0.102 (95% CI [0.069, 0.125]); and for “PBC ➔ PV ➔ BI,” it was 0.209 (95% CI [0.177, 0.256]). Furthermore, after controlling for the mediating variable (perceived value), the direct effects (c’) of the three antecedent variables on behavioral intention remained significant. This indicates that perceived value exerts a partial mediating role between the three core TPB variables and medical students’ SET intention. In summary, hypotheses H5a, H5b, and H5c were fully supported by the data.

## Discussion

Grounded in the specific context of high academic workloads and clinical pressures faced in global medical education, this study innovatively constructed and validated the “Extended Theory of Planned Behavior incorporating Perceived Value (Extended TPB)” framework. The empirical results not only confirmed the model’s extremely high predictive efficacy (explaining 95.9% of the variance in SET intention) but also profoundly revealed the core mediating and transformative mechanism of “Perceived Value” in medical students’ behavioral decision-making. By introducing the concept of value trade-offs from consumer behavior into educational psychology, this study provides a novel theoretical perspective for understanding and resolving the pervasive issues of “survey fatigue” and “perfunctory responses” in higher education.

### Theoretical extension: bridging the intention-behavior gap via psychological appraisal

The most significant theoretical contribution of this study is elucidating the explanatory limitations of the traditional Theory of Planned Behavior (TPB) in specific high-pressure contexts, and utilizing “perceived value” to achieve a theoretical upgrade. The classic TPB assumes that the more positive an individual’s attitude, the stronger the perceived social norm, and the higher the perceived behavioral control, the stronger their behavioral intention will be (Ajzen, 1991). However, this study argues that this assumption entails a hidden prerequisite: the individual must be in a state of “resource abundance.”

For medical students, overloaded medical curricula and rigorous clinical rotations render them inherently “time and cognitive resource poor.” Under such extreme conditions, traditional TPB antecedent variables often remain “dormant” and cannot directly or blindly translate into action. Our empirical data support this perspective: attitude, subjective norm, and perceived behavioral control must cross the psychological bridge of “perceived value” to exert a substantive impact on SET intention. The Extended TPB model proves that in high-pressure scenarios, medical students are no longer merely “norm compliers,” but rather highly rational “economic calculators.” They must engage in strict Return on Investment (ROI) calculations between limited energy (perceived costs) and substantive teaching improvements brought by SET (perceived benefits). The introduction of “perceived value” successfully fills the theoretical gap of traditional TPB in explaining “compromise behaviors” among highly burdened populations.

The classic TPB assumes a direct resource translation. However, in high-pressure learning environments, cognitive resources are finite. Our Extended TPB model contributes to educational psychology by demonstrating that external norms and internal attitudes must undergo a cognitive filtering process (Perceived Value) to activate behavioral intention.

### The “gatekeeper” effect and closed-loop feedback: cracking the “useless feedback” dilemma

This study revealed that perceived value exerts a primary predictor on medical students’ SET intention (*β* = 0.979, *p* < 0.001). This high standardized coefficient underscores that in high-demand educational contexts, student decision-making heavily mirrors rational choice and self-determined cognitive processes (Jia et al., 2025). When students perceive clear functional and developmental value, their autonomous motivation is activated, causing voluntary participation intention to surge exponentially and validating the integration of value-driven mechanisms into classical TPB models (Iskandar et al., 2024). This indicates that perceived value is the ultimate “gatekeeper” determining whether the evaluation behavior will actually occur. This finding provides robust data support to explain a longstanding pain point in international literature: low response rates triggered by the perception that feedback goes unvalued (Chen and Hoshower, 2003).

The exceptionally strong standardized coefficient (*β* = 0.979) and remarkably high explanatory power (*R^2^* = 95.9%) deserve a critical, nuanced discussion. While diagnostic tests (e.g., HTMT ratios) confirm statistical discriminant validity and rule out severe conceptual overlap, this extreme predictive power reflects a unique psychological reality among medical students experiencing burnout. In time-poor, high-stress academic settings, evaluation behavior is reduced to a binary cognitive heuristic: “Is this useful?” When students are cognitively depleted, their behavioral willingness becomes completely contingent on—and highly coupled with—their perceived value. Once a medical student acknowledges the “net utility” of the survey, the behavioral intention triggers automatically as a rational economic response. Thus, perceived value acts as an absolute psychological gatekeeper in this specific population. This also explains why intrinsic attitude (*β* = 0.498) exerted a substantially stronger impact on perceived value than external subjective norms (*β* = 0.141). For overburdened medical trainees, autonomous, internalized value perception is far more decisive in driving action than administrative coerciveness.

Currently, evaluation systems in many medical institutions globally are mired in a paradigm that “overemphasizes data collection while neglecting developmental feedback” (Winstone and Boud, 2022). This study emphasizes that if institutions fail to establish transparent “closed-loop feedback,” students will be unable to witness the substantive teaching changes resulting from their invested time and effort. When “perceived benefits” approach zero, even if the SET system (PBC) is highly user-friendly, students’ genuine intention to participate will plummet. The gatekeeper role of perceived value warns higher education administrators: the fundamental solution to perfunctory SET lies not in coercive mandates, but in institutions clearly demonstrating to students how evaluation results tangibly drive curriculum reform, thereby fundamentally rebuilding the “perceived utility” of evaluation tools.

### Internal drive outperforms external push: awakening students’ “feedback agency”

Among the antecedent variables in the Extended TPB model, the research results presented a highly revealing asymmetry in factor weights: attitude had the greatest impact on perceived value (*β* = 0.498), followed by perceived behavioral control (*β* = 0.333), while subjective norm, representing external environmental pressure, had the weakest impact (*β* = 0.141).

This stark contrast explicitly reflects the unique institutional and cultural characteristics of the Chinese medical education system introduced earlier. In highly hierarchical medical environments characterized by high power distance and Confucian work dynamism (Gao et al., 2025), students often perceive administrative mandates (subjective norms) merely as coercive bureaucratic tasks rather than genuine communicative opportunities. Consequently, external push alone generates minimal perceived utility, often defaulting to perfunctory rating behaviors rather than reflective feedback (Pineda and Ashour, 2022). Instead, our findings prove that to break through the utilitarian “compliance mindset” prevalent in Asian educational cultures (Fullerton et al., 2025), universities must awaken students’ intrinsic recognition of SET’s developmental value.

These empirical results directly challenge the traditional administrative-driven model of medical education management. Within the rigorous and often hierarchical culture of medical institutions, relying solely on prompting from academic a divisors or institutional mandates (subjective norms) often only yields “perfunctory compliance” or even “malicious grading” from students. As Nieminen et al. (2022) pointed out, modern educational assessment urgently needs to transcend the mere paradigm of “compliance.” This study robustly proves that only when students intrinsically recognize the positive significance of SET (internal attitude) and believe the process will not overly consume their precious time (behavioral control) can “value identification” be maximized. This process essentially empowers medical students, transitioning them from passive “data providers” to active stakeholders endowed with “student agency” in feedback. It verifies that within knowledge-intensive groups, stimulating intrinsic motivation is far more efficacious than exerting external administrative pressure. This resonates strongly with qualitative observations in clinical education that medical students will not meaningfully engage unless intrinsic value and personal agency are mobilized under high-strain training environments (Mokhtary et al., 2025).

These findings strongly resonate with Self-Determination Theory (SDT) (Ryan and Deci, 2000). The robust impact of internal attitude and perceived value, contrasted with the weak effect of subjective norm, highlights that SET behavior driven by autonomous motivation (valuing the task itself) is far more potent than controlled motivation (complying with external administrative pressure). True ‘feedback agency’ emerges only when students feel psychologically empowered rather than administratively coerced.

### The “cognitive cost” crisis: the practical trade-off during clinical rotations

Combined with independent samples testing, this study identified significant group heterogeneity in medical students’ SET intentions. Notably, senior students who are about to enter or are currently undergoing heavy clinical rotations exhibited the lowest SET intention.

Placing this phenomenon within the Extended TPB framework essentially reflects a severe “Perceived Behavioral Control (PBC) crisis” faced by senior medical students. As they advance to the clinical stage, their cognitive load surges under clinical service strain, time poverty, and hierarchical barriers (Fullerton et al., 2025), and traditional, lengthy questionnaires are perceived as a form of profitless “uncompensated labor” (Kreitzer and Sweet-Cushman, 2022). The sharp increase in time costs directly diminishes net perceived value, thereby triggering “survey fatigue.” This empirical outcome highly aligns with the model’s theoretical deductions and raises an urgent practical demand for medical institutions: for senior students (especially those in clinical rotations), there is a pressing need to abandon rigid, lengthy questionnaires in favor of contextualized, mobile-based “micro-evaluations.” This will effectively reduce the “operational costs” of participation and sustain the perceived value of the SET system. Evidence from recent educational interventions demonstrates that targeted strategies supporting workload management and student agency significantly enhance feedback utilization and reflective engagement among medical trainees (Harvey et al., 2025).

Based on the Importance-Performance prioritization, university administrators should strategically target Perceived Behavioral Control (PBC) as the primary intervention leverage. Since PBC exhibited high importance (*β* = 0.333) but the lowest performance score (3.845), institutions must focus on drastically reducing the “operational costs” of participation. For senior clinical trainees (the lowest-intention subgroup), rigid 40-item questionnaires should be replaced with mobile-based “micro-evaluations” (2–3 concise items post-shift). Additionally, establishing transparent “You Said, We Did” public dashboards allows students to directly witness how their evaluations drive clinical curriculum reform, fundamentally elevating net perceived value across diverse student profiles.

## Conclusion and limitations

Although this study has achieved certain theoretical extensions and empirical validation, some limitations remain, providing avenues for future research:

### Limitations of sample source

The data for this study were sourced primarily from a single medical university. Although the sample size is substantial (*N* = 3,365) and covers various majors and grades, the results may be influenced by the specific campus culture and management systems of that institution. Future research should consider conducting multi-center, cross-regional investigations across various medical institutions to verify the external validity and generalizability of the Extended TPB model across different tiers and types of medical higher education contexts.

### Cross-sectional nature of data and methodological limitations

First, this study relied on cross-sectional survey data, meaning the strong path relationships modeled (e.g., between Perceived Value and Behavioral Intention) reflect predictive associations rather than verified longitudinal causality. Second, although diagnostic tests confirmed discriminant validity and minimal common method bias, relying on self-reported measures may introduce potential social desirability bias or conceptual overlap between highly correlated constructs. Future research should adopt longitudinal or experimental designs and incorporate objective behavioral log data from university learning systems (e.g., actual login timestamps, evaluation completion rates, and qualitative feedback word counts) to bridge the intention-behavior gap and validate the model’s predictive precision over time.

### Space for theoretical model extension

While introducing perceived value significantly improved model explanatory power, other potential variables may still play a role in the complex medical school environment. Examples include a lack of psychological safety caused by anonymity concerns, or the degree of intimacy in the student-teacher relationship (e.g., the difference between one-on-one clinical mentoring and large lectures). Future research could attempt to incorporate “psychological safety” or “student-teacher trust” as moderating variables in the Extended TPB model, or combine qualitative methods such as in-depth interviews to comprehensively and multidimensionally dissect the psychological trajectories and decision-making mechanisms of medical students during the evaluation process.

## Data Availability

The raw data supporting the conclusions of this article will be made available by the authors, without undue reservation.

## References

[ref1] Adams M. J. D. Umbach P. D. (2012). Nonresponse and online student evaluations of teaching: understanding the influence of salience, fatigue, and academic environments. Res. High. Educ. 53, 576–591. doi: 10.1007/s11162-011-9240-5

[ref2] Ajzen I. (1991). The theory of planned behavior. Organ. Behav. Hum. Decis. Process. 50, 179–211. doi: 10.1016/0749-5978(91)90020-T

[ref3] Allison N. (2025). SETs (student evaluation of teaching): lessons to learn from differing biases of Chinese and UK students. Open Scholarsh. Teach. Learn. 5:134. doi: 10.56230/osotl.134

[ref4] Al-Maamari F. (2015). Response rate and teaching effectiveness in institutional student evaluation of teaching: a multiple linear regression study. High. Educ. Stud. 5, 9–20. doi: 10.5539/hes.v5n6p9

[ref5] Avery R. J. Bryant W. K. Mathios A. Kang H. Bell D. (2006). Electronic course evaluations: does an online delivery system influence student evaluations? J. Econ. Educ. 37, 21–37. doi: 10.3200/JECE.37.1.21-37

[ref9001] Bagozzi R. P. Yi Y. (1988). On the evaluation of structural equation models. Journal of the Academy of Marketing Science, 16, 74–94. doi: 10.1007/BF02723327

[ref6] Chen Y. Hoshower L. B. (2003). Student evaluation of teaching effectiveness: an assessment of student perception and motivation. Assess. Eval. High. Educ. 28, 71–88. doi: 10.1080/02602930301683

[ref9002] Cohen J. (1988). Statistical Power Analysis for the Behavioral Sciences (2nd ed.). Hillsdale, NJ: Lawrence Erlbaum Associates. doi: 10.4324/9780203771587

[ref9003] Davis F. D. (1989). Perceived usefulness, perceived ease of use, and user acceptance of information technology. MIS Quarterly, 13, 319–340. doi: 10.2307/249008

[ref7] Edgar S. Gibson W. (2016). Student feedback on learning and teaching: the value of focus groups. Focus Health Prof. Educ. Multi-Prof. J. 17, 81–84. doi: 10.3316/informit.245785968092513

[ref8] El Hassan K. (2009). Investigating substantive and consequential validity of student ratings of instruction. High. Educ. Res. Dev. 28, 319–333. doi: 10.1080/07294360902839917

[ref9004] Fornell C. Larcker D. F. (1981). Evaluating structural equation models with unobservable variables and measurement error. Journal of Marketing Research, 18, 39–50. doi: 10.1177/002224378101800104

[ref9] Fullerton P. D. Nguyen V. Pallath V. Sarkar M. (2025). Cultural influences on Asian health profession trainees seeking and receiving feedback: a scoping review. BMC Med. Educ. 26:30. doi: 10.1186/s12909-025-08307-z, 41318514 PMC12781924

[ref10] Gao X. Liu Y. Wang C. (2025). Cultural foundations of teacher occupational health: the impact of confucian work dynamism on burnout through grit among Chinese teachers. BMC Psychol. 13:390. doi: 10.1186/s40359-025-02724-x, 40241197 PMC12004800

[ref11] Groves R. M. (2005). Survey Errors and Survey Costs. Hoboken, NJ: John Wiley & Sons.

[ref9005] Hair J. F. Black W. C. Babin B. J. Anderson R. E. (2010). Multivariate Data Analysis (7th ed.). Englewood Cliffs, NJ: Prentice Hall.

[ref12] Harvey C.-J. Leedham-Green K. E. Koppel C. Maini A. Smith S. F. Morrell M. J. . (2025). Improving medical students’ learning strategies, management of workload and wellbeing: a mixed methods case study in undergraduate medical education. BMC Med. Educ. 25:606. doi: 10.1186/s12909-025-07118-6, 40275244 PMC12023436

[ref9006] Hoel A. Dahl T. I. (2019). Why bother? Student motivation to participate in student evaluations of teaching. Assessment \u0026amp; Evaluation in Higher Education, 44, 361–378. doi: 10.1080/02602938.2018.1511969

[ref13] Iskandar A. S. Wirawan H. Salam R. (2024). Students’ perceived value in higher education institutions: investigating the role of antecedents and context. Cogent Bus. Manag. 11:2313789. doi: 10.1080/23311975.2024.2313789

[ref14] Jia Q. Martínez-Hernández C. Peña-Martínez J. (2025). Mapping the integration of theory of planned behavior and self-determination theory in education: a scoping review on teachers’ behavioral intentions. Educ. Sci. 15:1529. doi: 10.3390/educsci15111529

[ref15] Kite M. E. Subedi P. C. Bryant-Lees K. B. (2015). Students’ perceptions of the teaching evaluation process. Teach. Psychol. 42, 307–314. doi: 10.1177/0098628315603062

[ref16] Kreitzer R. J. Sweet-Cushman J. (2022). Evaluating student evaluations of teaching: a review of measurement and equity bias in SETs and recommendations for ethical reform. J. Acad. Ethics 20, 73–84. doi: 10.1007/s10805-021-09400-w

[ref17] Macfadyen L. P. Dawson S. Prest S. Gašević D. (2016). Whose feedback? A multilevel analysis of student completion of end-of-term teaching evaluations. Assess. Eval. High. Educ. 41, 821–839. doi: 10.1080/02602938.2015.1044421

[ref18] Marlin J. W. (1987). Student perception of end-of-course evaluations. J. High. Educ. 58, 704–716. doi: 10.1080/00221546.1987.11778294, 37339054

[ref19] Min Q. Baozhi S. (1998). Factors affecting students’ classroom teaching evaluations. Teach. Learn. Med. 10, 12–15. doi: 10.1207/S15328015TLM1001_3

[ref20] Mokhtary F. Faghihi E. Sanagoo A. Jouybari L. (2025). You won’t learn until you want to: medical students’ experiences of the educational nature of the clinical environment. BMC Med. Educ. 25:661. doi: 10.1186/s12909-025-07211-w, 40329310 PMC12057205

[ref21] Nieminen J. H. Tai J. Boud D. Henderson M. (2022). Student agency in feedback: beyond the individual. Assess. Eval. High. Educ. 47, 95–108. doi: 10.1080/02602938.2021.1887080

[ref22] Nulty D. D. (2008). The adequacy of response rates to online and paper surveys: what can be done? Assess. Eval. High. Educ. 33, 301–314. doi: 10.1080/02602930701293231

[ref23] O’Donovan R. (2023). Missing the forest for the trees: investigating factors influencing student evaluations of teaching. Assess. Eval. High. Educ. 49, 453–470. doi: 10.1080/02602938.2023.2266862

[ref24] Pineda P. Ashour S. (2022). Student evaluation of teaching and student centeredness in the Humboldtian and Emirati tribal traditions of higher education. High. Educ. Res. Dev. 42, 1732–1747. doi: 10.1080/07294360.2022.2156483

[ref25] Porter S. R. Umbach P. D. (2006). Student survey response rates across institutions: why do they vary? Res. High. Educ. 47, 229–247. doi: 10.1007/s11162-005-8887-1

[ref26] Royal K. D. Temple L. J. Neel J. A. Nelson L. L. (2018). Psychometric validation of a medical and health professions course evaluation questionnaire. Am. J. Educ. Res. 6, 38–42. doi: 10.12691/education-6-1-6

[ref27] Ryan R. M. Deci E. L. (2000). Self-determination theory and the facilitation of intrinsic motivation, social development, and well-being. Am. Psychol. 55, 68–78. doi: 10.1037/0003-066X.55.1.68, 11392867

[ref28] Shah M. Pabel A. (2019). Making the student voice count: using qualitative student feedback to enhance the student experience. J. Appl. Res. High. Educ. 12, 194–209. doi: 10.1108/JARHE-02-2019-0030

[ref29] Sigurdardottir M. S. Rafnsdottir G. L. Jónsdóttir A. H. Kristofersson D. M. (2022). Student evaluation of teaching: gender bias in a country at the forefront of gender equality. High. Educ. Res. Dev. 42, 954–967. doi: 10.1080/07294360.2022.2087604, 37339054

[ref30] Spooren P. Brockx B. Mortelmans D. (2013). On the validity of student evaluation of teaching: The state of the art. Rev. Educ. Res. 83, 598–642. doi: 10.3102/0034654313496870

[ref31] Spoorena P. Van Loon F. (2012). Who participates (not)? A non-response analysis on students’ evaluations of teaching. Proc. Soc. Behav. Sci. 69, 990–996. doi: 10.1016/j.sbspro.2012.12.025

[ref32] Uijtdehaage S. O’Neal C. (2015). A curious case of the phantom professor: mindless teaching evaluations by medical students. Med. Educ. 49, 928–932. doi: 10.1111/medu.12647, 26296409

[ref33] van Bruggen L. ten Cate O. Chen H. C. (2020). Developing a novel 4-C framework to enhance participation in faculty development. Teach. Learn. Med. 32, 371–379. doi: 10.1080/10401334.2020.1742124, 32251617

[ref34] Winstone N. E. Boud D. (2022). The need to disentangle assessment and feedback in higher education. Stud. High. Educ. 47, 656–667. doi: 10.1080/03075079.2020.1779687

[ref35] Winstone N. E. Carless D. (2021). Who is feedback for? The influence of accountability and quality assurance agendas on the enactment of feedback processes. Assess. Educ. Princ. Policy Pract. 28, 261–278. doi: 10.1080/0969594X.2021.1926221, 37339054

[ref36] Zabaleta F. (2007). The use and misuse of student evaluations of teaching. Teach. High. Educ. 12, 55–76. doi: 10.1080/13562510601102131

